# The Conserved G-Protein Coupled Receptor FSHR-1 Regulates Protective Host Responses to Infection and Oxidative Stress

**DOI:** 10.1371/journal.pone.0137403

**Published:** 2015-09-11

**Authors:** Elizabeth V. Miller, Leah N. Grandi, Jennifer A. Giannini, Joseph D. Robinson, Jennifer R. Powell

**Affiliations:** Department of Biology, Gettysburg College, Gettysburg, Pennsylvania, United States of America; CSIR-Central Drug Research Institute, INDIA

## Abstract

The innate immune system’s ability to sense an infection is critical so that it can rapidly respond if pathogenic microorganisms threaten the host, but otherwise maintain a quiescent baseline state to avoid causing damage to the host or to commensal microorganisms. One important mechanism for discriminating between pathogenic and non-pathogenic bacteria is the recognition of cellular damage caused by a pathogen during the course of infection. In *Caenorhabditis elegans*, the conserved G-protein coupled receptor FSHR-1 is an important constituent of the innate immune response. FSHR-1 activates the expression of antimicrobial infection response genes in infected worms and delays accumulation of the ingested pathogen *Pseudomonas aeruginosa*. FSHR-1 is central not only to the worm’s survival of infection by multiple pathogens, but also to the worm’s survival of xenobiotic cadmium and oxidative stresses. Infected worms produce reactive oxygen species to fight off the pathogens; FSHR-1 is required at the site of infection for the expression of detoxifying genes that protect the host from collateral damage caused by this defense response. Finally, the FSHR-1 pathway is important for the ability of worms to discriminate pathogenic from benign bacteria and subsequently initiate an aversive learning program that promotes selective pathogen avoidance.

## Introduction

To survive an attack by a microbial pathogen, a host must first recognize that the pathogen is attempting to cause an infection so that it can then mount a response. Recognition can occur via direct binding of host pattern-recognition receptors (PRRs), including Toll-like receptors (TLRs) and NOD-like receptors (NLRs), to microbe-associated molecular patterns (MAMPs) [1]. Direct pathogen recognition is an effective detection mechanism for professional immune cells that exist in normally sterile environments: any microbe is a threat that therefore necessitates a response. In contrast, epithelial cells commonly encounter microbes of the normal microbiota that are benign or even beneficial to the host. Direct recognition of MAMPs is not sufficient for the discrimination of pathogen vs. non-pathogen because these broadly conserved molecules are found among both pathogenic and non-pathogenic members of a microbial group. The ability to differentiate a pathogen from a non-pathogen is critical because inappropriate activation of the innate immune response is energetically wasteful, can harm the beneficial microbiota, and can non-specifically harm the host itself [2].

Recognition of the “patterns of pathogenesis” associated with an infection instead of or in addition to recognition of the microbe itself permits the host innate immune system to distinguish between a pathogen and non-pathogen [2]. One such pattern of pathogenesis is the disruption of core host processes by microbial toxins. For example, *Pseudomonas aeruginosa* Exotoxin A cripples translation elongation by ADP-ribosylating EF-2. The *Caenorhabditis elegans* bZip transcription factor ZIP-2 is only translated when general translation is impeded by Exotoxin A; when expressed, ZIP-2 induces the transcription of a collection of antimicrobial innate immune effectors [3]. Thus, *C*. *elegans* uses ZIP-2-mediated surveillance of translation to detect a *P*. *aeruginosa* infection and activate the appropriate defense response.

Damage-Associated Molecular Patterns (DAMPs) that reveal physical or chemical damage to host cells can also be sensed by the host immune system as a pattern of pathogenesis and thus as a mode of discriminating pathogens from non-pathogens. For example, the tyrosine metabolite 4-hydroxyphenyllactic acid (HPLA) is a DAMP produced by *C*. *elegans* in response to sterile wounding or infection with the fungus *Drechmeria coniospora*. HPLA is a ligand of the G-protein coupled receptor (GPCR) DCAR-1, which subsequently activates a MAPK-mediated defense response [4]. Thus, this GPCR does not bind the pathogen directly; rather, it senses damage caused by a pathogen but not a non-pathogen.

GPCR-based innate immune recognition of bacterial pathogens extends beyond the DCAR-1/HPLA system. Bacterial uracil, which is secreted at higher levels by pathogens than by commensals, is a pattern of pathogenesis that is recognized by an as-yet-unidentified GPCR in the *Drosophila melanogaster* gut epithelium. Uracil-activated GPCR signaling triggers the production of reactive oxygen species (ROS), an essential component of the *Drosophila* defense against midgut infections [5,6]. In mice, nasal epithelial cells detect acyl-homoserine lactone quorum sensing molecules, which are associated with virulence in Gram-negative bacteria, via GPCR/phospholipase C-mediated signaling [7]. These chemosensory cells then activate a local inflammatory response [8]. These examples illustrate that G-protein coupled receptors, long known to play important roles in diverse processes, are emerging as central players in the detection of infection.

Here we describe a conserved GPCR that acts in the intestine to regulate protective host responses toward toxic xenobiotics and ingested pathogens. The *C*. *elegans* Follicle Stimulating Hormone Receptor homolog FSHR-1 is required for the innate immune response to diverse pathogens [9]. FSHR-1 delays the intestinal accumulation of ingested pathogenic bacteria. Upon infection, the FSHR-1 pathway activates not only the expression of known infection response genes, but also a collection of stress response genes. FSHR-1 protects worms from toxic reactive oxygen species (ROS) and cadmium, independent of infection. FSHR-1 is required for worms to learn aversive behavior toward pathogens, and overexpression causes avoidance of benign bacteria, indicating that the FSHR-1 pathway contributes to the worm’s ability to discriminate pathogen from non-pathogen.

## Results and Discussion

### FSHR-1 delays pathogen accumulation in the intestine

**Fig 1:**
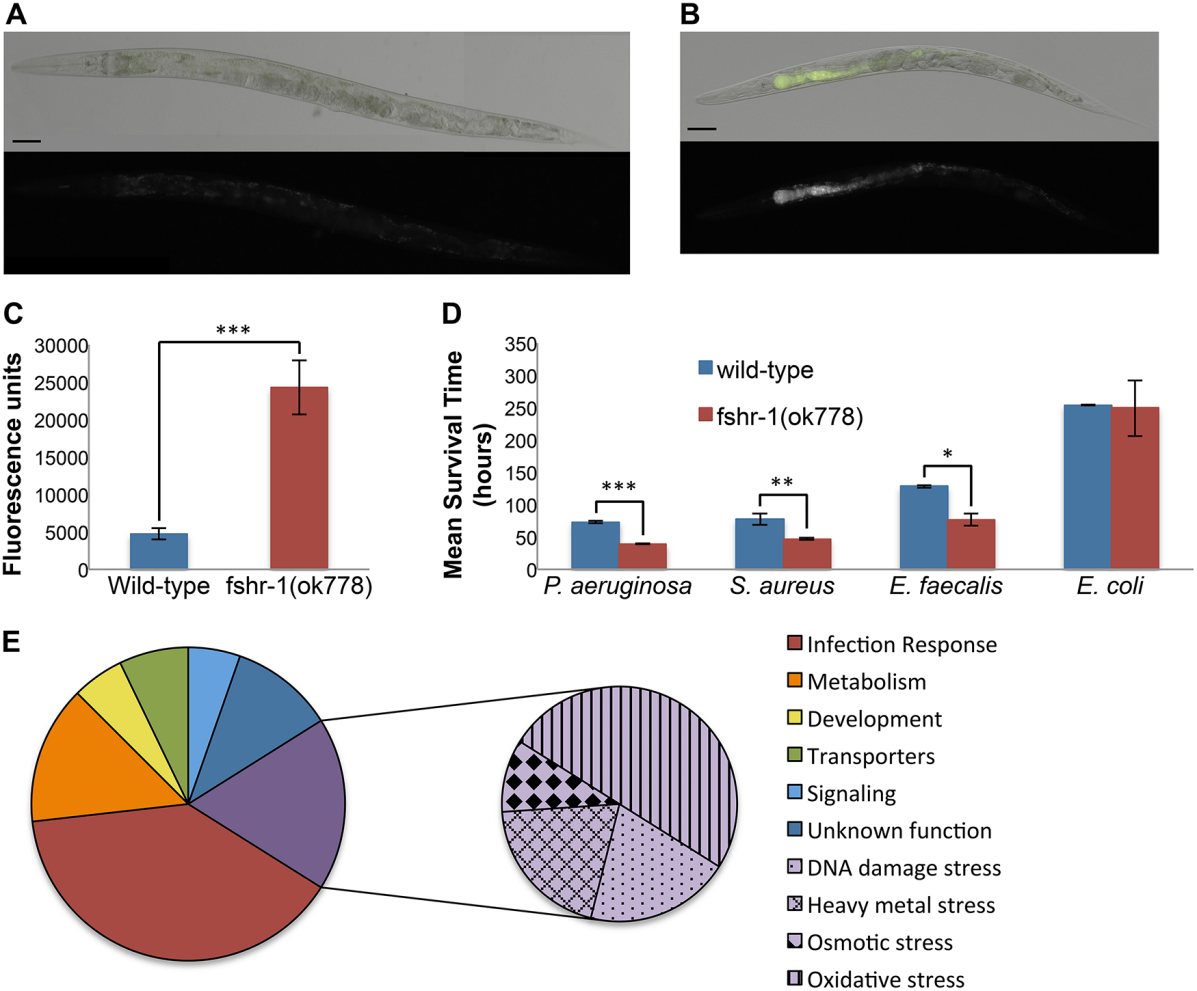
FSHR-1 is required for the defense against diverse pathogens. Representative wild-type (A) and *fshr-1(ok778)* mutant (B) worms fed GFP-PA14 for 18 hours. Scale bars = 100 μm. (C) Quantification of the sum of the intestinal fluorescence intensity for at least 10 representative individual worms per condition. (D) Mean survival time of wild-type and *fshr-1(ok778)* mutant worms exposed to different bacterial food sources. * P<0.05, **P<0.01, *** P<0.0001 in unpaired t-test. Error bars are SEM. (E) Functional annotation of genes whose mean expression from three microarray replicates in wild-type worms infected with PA14 for 4 hours was at least 2-fold higher than their mean expression in *fshr-1(ok778)* mutants infected with PA14 for 4 hours, with P<0.05. Genes known to be involved both in infection response and stress response were grouped with stress response.

Intestinal infection by *Pseudomonas aeruginosa* strain PA14 progresses more quickly in *fshr-1* mutants than in wild-type *C*. *elegans*. Wild-type worms exposed to PA14 initially appear healthy, but by 8 hours post-infection they begin to show early symptoms of the infection, including a distension of the lumen of their intestine. By 24 hours post-infection, the worms may appear sickly and slightly small and the intestine has begun to accumulate live actively dividing bacteria [10]. To determine the effect of a null *fshr-1(ok778)* mutation on this progression of infection, we exposed worms to GFP-labeled PA14. At the light microscopic level, *fshr-1(ok778)* mutant worms follow the same sequence of infection as wild-type worms, but develop symptoms of infection more quickly. For example, by 18 hours of exposure to GFP-labeled PA14, most wild-type worms have very little fluorescence in the lumen of their intestines, suggesting they have not yet amassed a substantial amount of PA14 (Fig 1A and 1C). In contrast, after 18 hours of exposure to GFP-PA14, *fshr-1(ok778)* mutants have already accumulated a significantly greater amount of the fluorescent pathogen in their intestinal lumens (P<0.0001) (Fig 1B and 1C).

Both wild-type and *fshr-1(ok778)* mutant worms eventually succumb to a PA14 infection. However, infected *fshr-1(ok778)* worms have a mean survival time of 39 hours, significantly less than the wild-type mean survival time of 73 hours (P<0.0001) (Fig 1D). We previously showed that the slope of the survival curves is approximately the same but the *fshr-1* survival curve is shifted to the left [9]. This observation, combined with the more rapid accumulation of pathogenic PA14, suggests that FSHR-1 protects *C*. *elegans* from bacterial infection by delaying the establishment or progression of the infection.

Could FSHR-1 be a pattern recognition receptor? Its extracellular domain contains seven Leucine-Rich Repeat domains, protein-protein interaction motifs that are also present in the ligand-binding domains of Toll-like receptors (TLRs) and Nod-like receptors (NLRs) [11]. However, *fshr-1(ok778)* mutants are more sensitive than wild-type worms to infection by diverse pathogens, including not only the Gram negative pathogen PA14, but also the Gram positive bacterial pathogens *Staphylococcus aureus* (P>0.01) and *Enterococcus faecalis* (P>0.05) (Fig 1D) [9], as well as the eukaryotic fungal pathogen *Candida albicans* (R. Pukkila-Worley and J. Powell, personal communication). Because these microbes are members of structurally dissimilar classes, it is unlikely that FSHR-1 detects infection by binding directly to a common MAMP possessed by these distinct phyla. Rather than serving as a direct PRR like fellow LRR-containing TLRs and NLRs, we propose that FSHR-1 is an indirect sensor of infection.

### FSHR-1 regulates the expression of immune and stress-associated genes

To understand the effect of the FSHR-1 pathway on the innate immune response in *C*. *elegans*, we examined gene expression in infected worms. We previously showed that FSHR-1 is required in *C*. *elegans* for the transcriptional induction of a set of known infection response genes upon exposure to PA14 [9]. Many of these genes are thought to have antimicrobial activity that antagonizes the infecting pathogen [12]. To gain a more complete picture of the downstream transcriptional impact of the FSHR-1 pathway, we performed a full-genome microarray on wild-type or *fshr-1(ok778)* null mutant worms that were either infected with PA14 or grown on relatively non-pathogenic *E*. *coli* OP50 as a control [13]. We used quantitative RT-PCR (S1 Fig) and transcriptional reporter (S2 Fig) analyses to validate the microarray results. Thirty four genes were expressed at greater than 2-fold higher levels (P<0.05) in wild-type worms relative to *fshr-1(ok778)* mutants grown on *E*. *coli* (S1 Table). Fifty-six genes were expressed at greater than 2-fold higher levels (P<0.05) in wild-type worms infected with PA14 relative to infected *fshr-1(ok778)* mutants (S2 Table). When we examined the reported functions of the genes whose expression in infected worms depends on *fshr-1*, we observed that many were known antimicrobial infection response genes; in addition, 18% of *fshr-1*-regulated genes were associated with some type of cellular stress (Fig 1E).

### FSHR-1 acts in the intestine to mediate survival of heavy metal and oxidative stress

One way that host cells sense an infection indirectly is by assessing infection-induced cellular damage or stress. Because the loss of *fshr-1* is associated with sensitivity to diverse pathogens and results in the misregulation of a set of stress-response genes, we hypothesized that the FSHR-1 pathway may contribute to the worm’s immune defenses by responding to general damage or cellular stress caused by infection. As a corollary, we predicted that the FSHR-1 pathway might also sense some types of cellular stress in the absence of infection. To test this prediction, we measured the survival of worms subjected to several types of stress.

**Fig 2:**
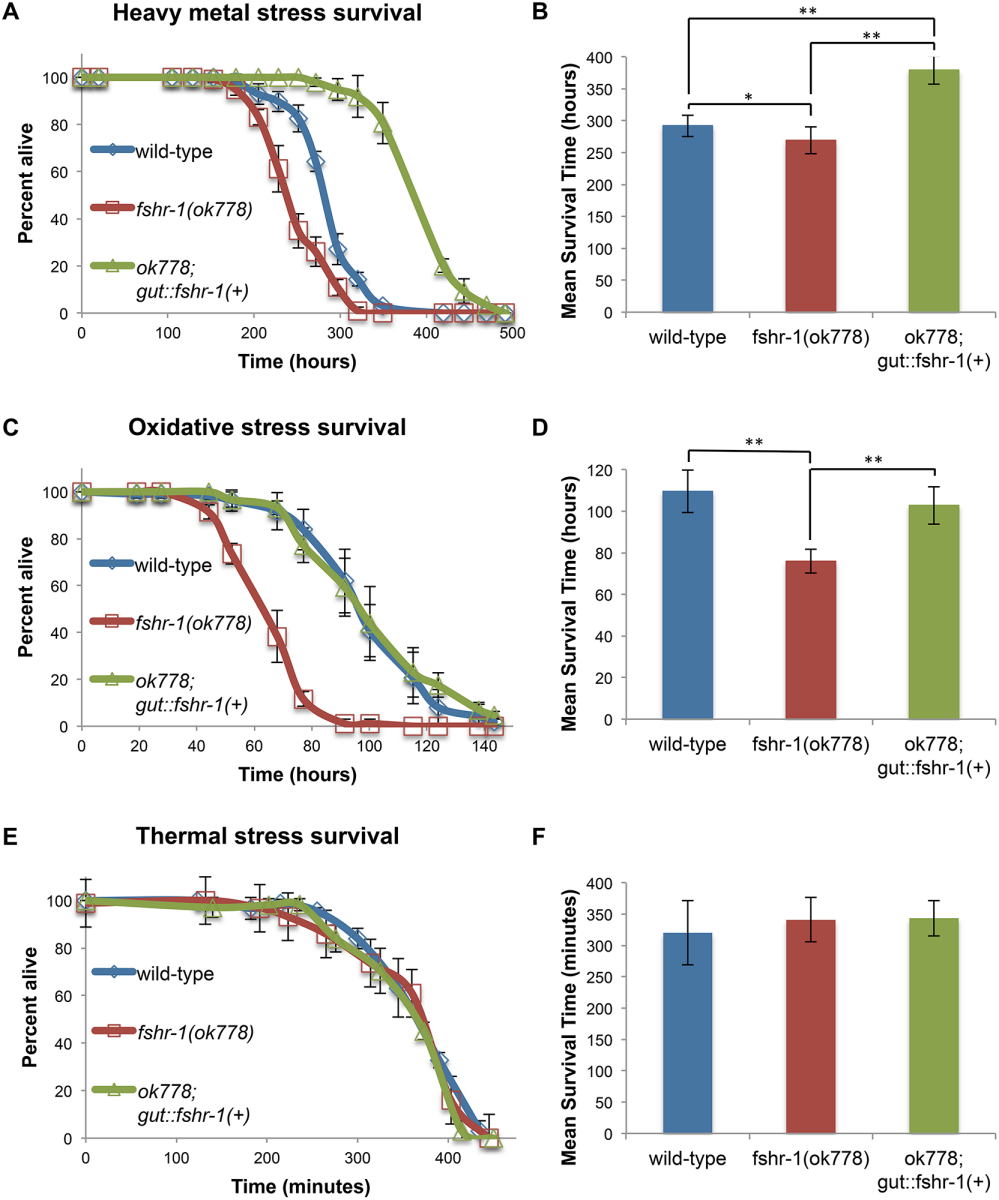
FSHR-1 is required for the survival of heavy metal and oxidative stress. Wild-type, *fshr-1(ok778)* mutants, and mutants rescued with *fshr-1(+)* in the intestine were measured for their ability to survive different types of stress. Worms were subjected to 100 μM cadmium (A, B), 5 mM paraquat (C, D), or 37°C (E, F). Representative survival curves for approximately 80–100 worms for each condition are shown in (A, C, and E) with standard deviation error bars. The mean survival time for each genotype was calculated for at least three independent experiments (B, D, F). Error bars are SEM. One-way ANOVA and Tukey HSD test, *P<0.05, **P<0.01.

To determine the effects of heavy metal stress on *fshr-1(ok778)* mutants, we exposed L4 worms to 100 μM cadmium and measured their survival. *fshr-1(ok778)* mutants died significantly more quickly in the presence of cadmium than wild-type worms (One-way ANOVA, F = 110.68; Tukey HSD test, P<0.05) (Fig 2A and 2B). Expression of *fshr-1* in the intestine is necessary and sufficient for its role in the response to infection by PA14 [9], so we also tested whether *fshr-1(+)* expressed in the intestine could rescue the cadmium sensitivity phenotype of *fshr-1(ok778)* mutants. Interestingly, expression of *fshr-1(+)* from an intestinal promoter rescued the cadmium sensitivity phenotype and conferred resistance to cadmium exposure (One-way ANOVA, F = 110.68; Tukey HSD test, P<0.01) (Fig 2A and 2B). These data suggest that the *fshr-1* pathway is not only required for surviving cadmium exposure, but also could be sufficient in the intestine to protect worms against cadmium-induced damage independent of infection.

To determine whether the *fshr-1* pathway plays a role in the survival of oxidative stress, we placed worms on plates containing 5 mM paraquat, which causes the production of reactive oxygen species (ROS). *fshr-1(ok778)* mutants died significantly more quickly than wild-type worms (One-way ANOVA, F = 40.96; Tukey HSD test, P<0.01) (Fig 2C and 2D). Additionally, intestinal expression of *fshr-1(+)* rescued the paraquat sensitivity phenotype (One-way ANOVA, F = 40.96; Tukey HSD test, P<0.01), suggesting that the *fshr-1* pathway is important for the ability of worms to survive infection-independent oxidative stress in the same tissue in which it acts to promote survival of infection.

Wild-type worms subjected to thermal stress survive at 37°C for an average of 320 minutes (Fig 2E and 2F). The mean survival of *fshr-1(ok778)* mutants at 37°C is not significantly different from the survival of wild-type worms (One-way ANOVA, F = 0.42, P = 0.669), indicating that the *fshr-1* pathway does not respond to the damage caused by high heat.

Because *fshr-1(ok778)* mutants are sensitive to oxidative and cadmium stress but not to thermal stress, the FSHR-1 pathway responds specifically to certain types of stress rather than more broadly to any type of cellular damage. Many studies have shown that cadmium exposure causes oxidative stress through the production of ROS (recently reviewed in [14]). Microarray experiments have also shown that cadmium exposure induces many of the same changes in gene expression as infection by *Pseudomonas aeruginosa* [12]. It is possible this common response is due to the fact that ROS play important roles in the interaction between host and pathogen during an infection [15]. Thus, host cells are subjected to oxidative stress both when infected and when exposed to toxic doses of cadmium. We propose a model in which the FSHR-1 pathway senses and responds to oxidative damage from a variety of sources, including exogenously added chemicals and the worm’s own immune system.

### FSHR-1 controls the induction of the oxidative-stress response gene *gcs-1* in response to infection

*C*. *elegans* intestinal cells generate an oxidative burst upon infection with *Enterococcus faecalis* strain OG1RF [15]. The dual oxidase Ce-Duox1/BLI-3 is responsible for the formation of these ROS, and is required for the worm to fight the *E*. *faecalis* infection [16,17]. Because ROS react with and damage a variety of biological molecules, and do not discriminate against host or pathogen targets, *C*. *elegans* intestinal cells and other host cells that produce ROS as a form of pathogen defense must also induce an oxidative stress response to detoxify the ROS and protect itself from collateral damage. In worms infected with *E*. *faecalis* or PA14, this detoxification response is mediated by the Nrf transcription factor SKN-1 [18].

**Fig 3:**
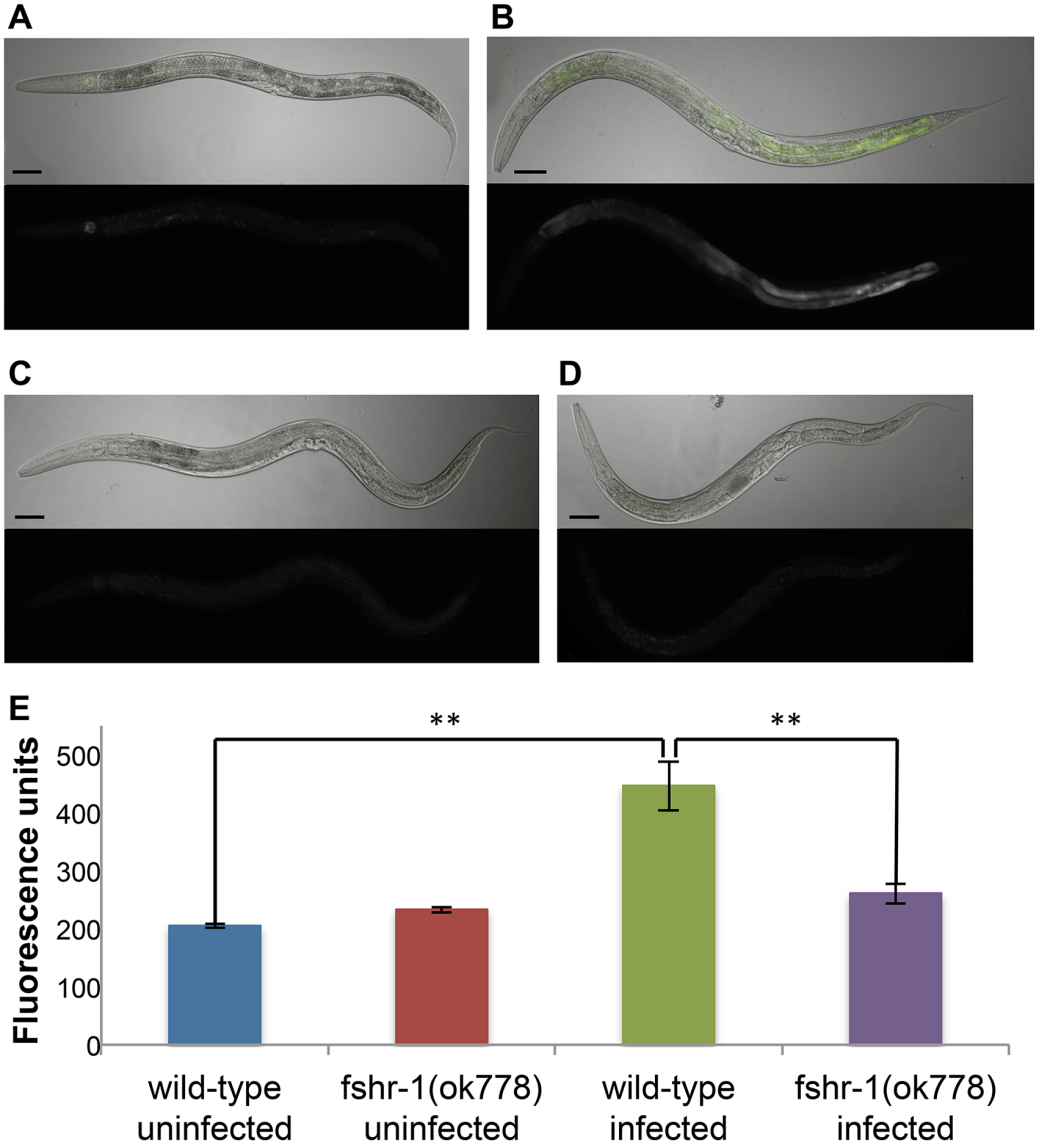
FSHR-1 is required for the induction of the oxidative stress response by PA14. *ldIs3[gcs-1*::*gfp]* (A, B) or *fshr-1(ok778); ldIs3[gcs-1*::*gfp]* (C, D) worms were exposed to OP50 (A, C) or PA14 (B, D) for 5 hours. Representative worms show that in a wild-type background, *gcs-1*::*gfp* is induced by pathogen exposure; this induction depends on *fshr-1*. Scale bars = 100 μm. (E) Quantification of the mean fluorescence intensity of the intestine for each condition (see Methods). Error bars are SEM. One-way ANOVA and Tukey HSD test, **P<0.01.

GCS-1 is a phase II detoxification enzyme and catalyzes the rate-limiting step of glutathione biosynthesis. It is an important target of SKN-1 and is a participant in the oxidative stress response in *C*. *elegans*. Wild-type worms induce the intestinal expression of a *gcs-1*::*gfp* transcriptional reporter within 5 hours of infection with PA14 (One-way ANOVA, F = 13.33; Tukey HSD test, P<0.01)(Fig 3A, 3B and 3E). Note that this change in gene expression occurs rapidly, before visible physical symptoms appear. In contrast, *fshr-1(ok778)* mutant worms fail to induce expression of *gcs-1*::*gfp* upon PA14 infection (Fig 3C–3E). These data, in combination with the microarray (Fig 1E), indicate that *fshr-1* is required for the early activation of the host’s protective oxidative stress response upon infection.

### FSHR-1 mediates the avoidance of pathogens

**Fig 4:**
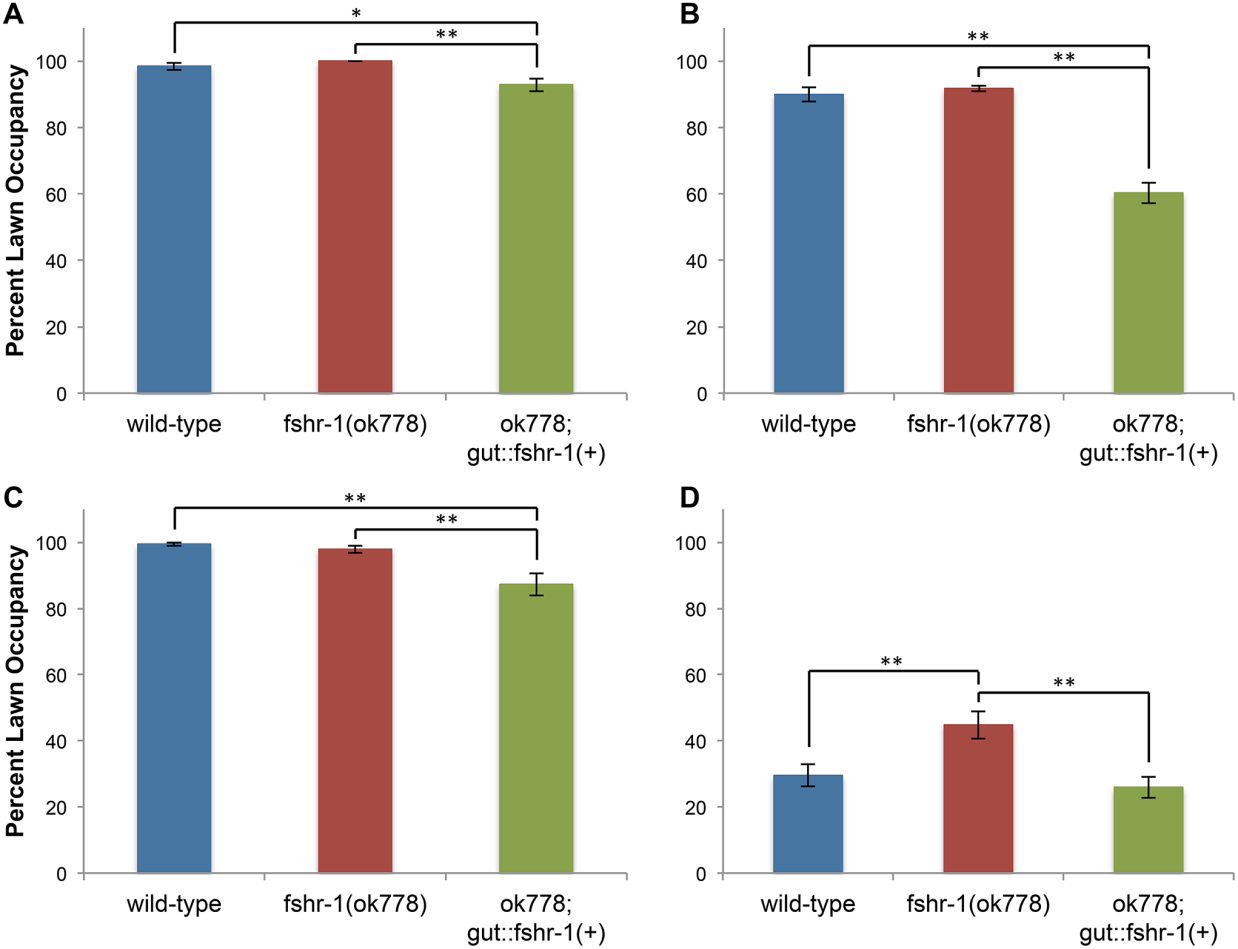
FSHR-1 promotes the avoidance of pathogenic PA14. The occupancy of wild-type, *fshr-1(ok778)*, and *ok778; agIs35[gut*::*fshr-1(+)]* worms on a lawn of relatively non-pathogenic *E*. *coli* OP50 (A, B) or pathogenic *P*. *aeruginosa* PA14 (C, D) was measured <1 hour (A, C) and 9 hours (B, D) after transferring worms to the lawn. Error bars are SEM. One-way ANOVA and Tukey HSD test, *P<0.05, **P<0.01.

Another important component of immunity is a behavioral response to pathogen exposure. Worms increase their survival when they avoid a lawn of pathogenic bacteria, presumably because they ingest fewer pathogen cells [19–22]. To determine whether *fshr-1* contributes to the behavioral avoidance of pathogens, we measured the occupancy of worms on lawns of pathogenic and non-pathogenic bacteria. Both wild-type and *fshr-1(ok778)* mutant worms prefer to forage on a lawn of relatively non-pathogenic *E*. *coli* strain OP50, with more than 90% of the worms found occupying the lawn (Fig 4A and 4B). Wild-type worms can distinguish between pathogenic and non-pathogenic bacteria and initiate an aversive learning program to actively avoid feeding on the pathogen [20,23,24]. Initially, wild-type worms are attracted to pathogenic PA14 and occupy a lawn at levels similar to a non-pathogenic OP50 lawn (Fig 4A and 4C). After 9 hours of exposure to PA14, however, only 29% of wild-type worms occupy a lawn of the pathogen (Fig 4D). Although *fshr-1(ok778)* mutants are indistinguishable from wild-type in their occupancy of non-pathogenic OP50 lawns and of their initial occupancy of pathogenic PA14 lawns (Fig 4A, 4B and 4C), *fshr-1(ok778)* mutants are impaired in their ability to learn pathogen avoidance. 45% of *fshr-1(ok778)* mutants remain on a lawn of PA14 after 9 hours of exposure (One-way ANOVA, F = 8.89; Tukey HSD test, P<0.01) (Fig 4D). Additionally, *fshr-1*(RNAi) worms show significantly lower lawn occupancy (51%) after 9 hours of exposure to PA14 than control RNAi worms (21%, two-sample T-test, P<0.001), confirming that FSHR-1 is required for the ability of worms to avoid pathogenic bacteria (S3 Fig).

Learned behavioral avoidance requires that the worm identify the microbe and associate that identity with the effects of the infection. Microbial identification is mediated by a chemosensory circuit that detects secondary metabolites and activates a neuroendocrine signaling pathway [23,24]. Although the detection of secondary bacterial metabolites associated with virulence can indicate not only the presence of a bacterium but also an infection, it is likely this neuronal signal is integrated with signals from the site of infection. To determine whether FSHR-1 acts in the intestine to influence pathogen avoidance, we measured the lawn occupancy of worms expressing FSHR-1 exclusively in the intestine. Intestine-specific expression of the wild-type *fshr-1(+)* from the integrated transgene *agIs35[gut*::*fshr-1(+)]* was sufficient to rescue the PA14 avoidance defect of *fshr-1(ok778)* null mutants (One-way ANOVA, F = 8.89; Tukey HSD test, P<0.01), suggesting that FSHR-1 does function in the intestine for this role (Fig 4D).

Interestingly, worms with the *agIs35[gut*::*fshr-1(+)]* transgene inappropriately avoid bacterial lawns in the absence of a pathogen. Only 60% of *gut*::*fshr-1(+)* transgenic worms occupy a lawn of OP50 after 9 hours of exposure, while 90% of wild-type and *fshr-1(ok778)* mutant worms are present in the lawn (Fig 4B) (One-way ANOVA, F = 54.84, P<0.0001). Even in the absence of aversive learning, naïve transgenic worms show a modest but statistically significant 10% reduction in their occupancy of a bacterial lawn on which they were recently placed (Fig 4A and 4C) (OP50: One-way ANOVA, F = 8.38; Tukey HSD test, P<0.01. PA14: One-way ANOVA, F = 9.85; Tukey HSD test, P<0.01). It is possible that the heterologous intestinal promoter and multiple copies in which the *gut*::*fshr-1(+)* transgene is present cause a hyper-activation of the FSHR-1 pathway, which could incorrectly or constitutively signal the need to avoid a microbe.

**Fig 5:**
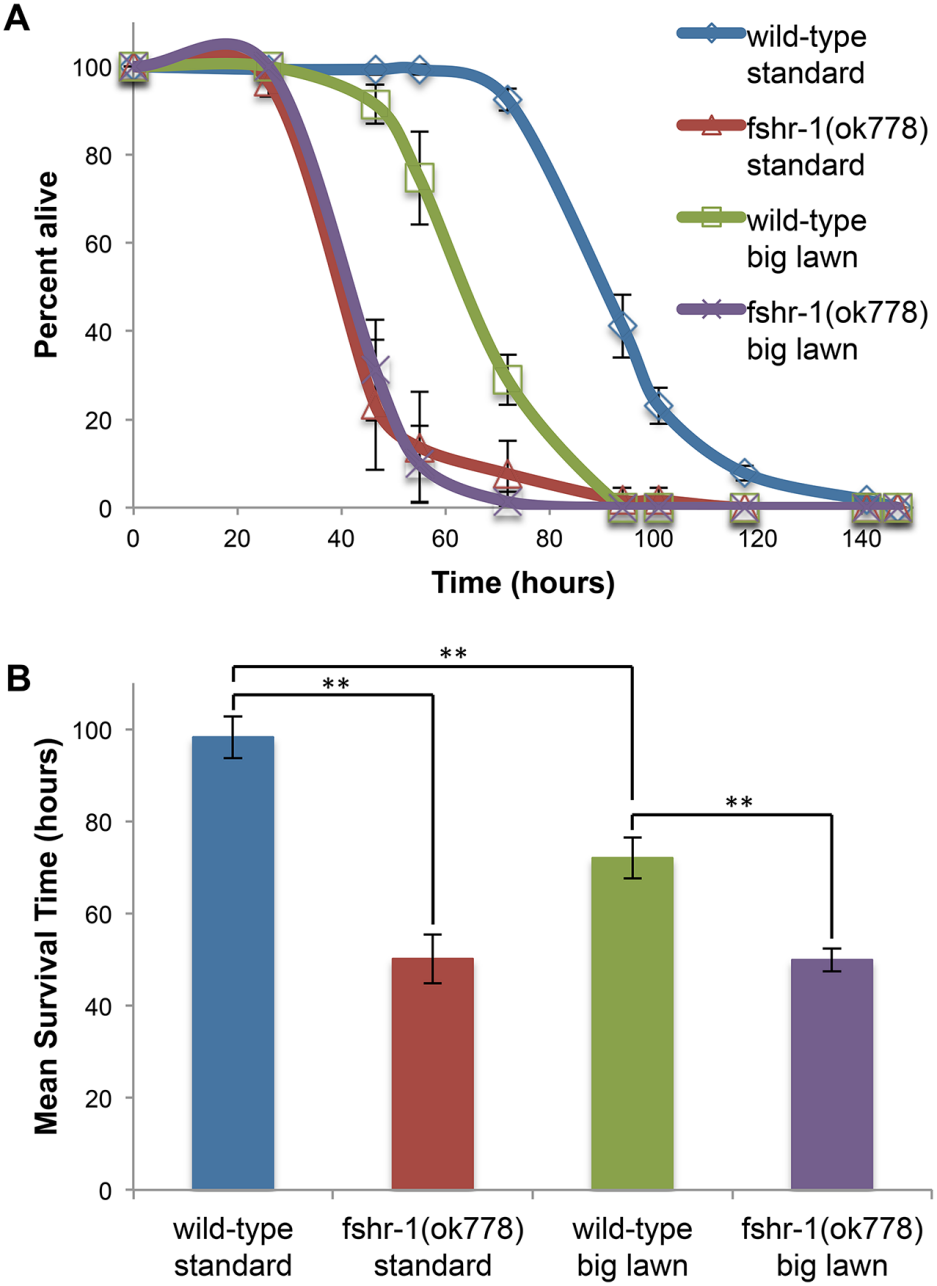
Pathogen avoidance contributes to the pathogen sensitivity of *fshr-1* mutants. The survival of worms was measured when exposed to a standard small lawn of PA14 or a big lawn that covered the entire surface of the agar. (A) Representative killing assay. (B) Mean survival time was measured and compared using one-way ANOVA and Tukey HSD test, **P<0.01. Error bars are standard deviation (A) or SEM (B).

To determine the extent to which an altered avoidance behavior contributes to the infection sensitivity phenotype of *fshr-1(ok778)* mutants, we performed killing assays using standard or big lawns. In a standard killing assay, the lawn of pathogen covers only part of the plate, giving the worms the option to avoid this lawn and reduce their consumption of the pathogen. A big lawn assay removes this choice by covering the entire surface of the agar with the pathogen. Wild-type worms die more quickly in a big-lawn killing assay than in a standard small-lawn killing assay (One-way ANOVA, F = 255.04; Tukey HSD test, P<0.01), confirming previous reports that avoiding the lawn is one successful strategy for fighting infection (Fig 5A and 5B) [19,25,26]. Infected *fshr-1(ok778)* mutants die along a nearly identical time course in standard small- and big-lawn killing assays, providing additional support for the conclusion that the FSHR-1 pathway regulates a behavioral pathogen avoidance component of the immune system. Importantly, *fshr-1(ok778)* mutants are still more sensitive to infection than wild-type worms in a big-lawn killing assay (One-way ANOVA, F = 255.04; Tukey HSD test, P<0.01) (Fig 5A and 5B), indicating that regulation of pathogen avoidance is not the only contribution of the FSHR-1 pathway to innate immune defense.

## Conclusions

In summary, the conserved GPCR FSHR-1 mediates multiple defensive and protective facets of the *C*. *elegans* innate immune system. FSHR-1 is required for the survival both of infection by diverse pathogens and of xenobiotic insults that cause oxidative stress. Upon infection, FSHR-1 activates expression of a collection of known antimicrobial infection response genes that defend the worm by attacking the pathogen. FSHR-1 also activates expression of a collection of oxidative stress response genes that detoxify ROS produced as part of the worm’s defense and thereby protect the worm against oxidative damage. FSHR-1 is additionally required for the protective ability of worms to sense an infection and modify their behavior to avoid pathogens.

We have previously shown that FSHR-1 is necessary and sufficient in the intestine for its role in promoting survival of infection by ingested pathogens [9]. Here we provide evidence that this receptor coordinates multiple facets of innate immunity from the intestine, the site of infection. An intestinal role for FSHR-1 in the response to infection-induced oxidative stress is not unexpected because the worms must sense and respond to oxidative damage at the site of ROS production, the intestine. FSHR-1 also acts in the intestine for its protective role against infection-independent oxidative stress, suggesting the same mechanism can be used to sense xenobiotic ROS production despite the fact that the worm’s exposure to ROS-generating cadmium and paraquat is not restricted to the intestinal epithelium.

Learned pathogen avoidance behavior is a more complex phenomenon that requires the integration of sensory signals from multiple tissues. ASJ chemosensory neurons detect secondary metabolites produced by bacteria via a G-protein signaling pathway and communicate with interneurons via a TGF-β neuroendocrine signal [26]. The intestine and hypodermis sense perturbations in essential cellular processes caused by bacterial toxins at the site of an infection [27]. The bacterial identity signal from the neurons is integrated with an infection signal from the intestine and/or hypodermis to modulate the worm’s behavior and promote learned avoidance of a pathogen via a suppression of aerotaxis and activation of serotonin signaling [23,24,27]. Although G-protein signaling is important for the neuronal identification of bacteria, and it is a formal possibility that FSHR-1 also acts in the neurons, our data indicate that the GPCR FSHR-1 acts in the intestine to promote the avoidance of pathogens, suggesting that FSHR-1 contributes to the intestinal detection of infection rather than the neuronal identification of bacteria.

How might the FSHR-1 pathway sense an infection? The production of ROS by host cells is a widely utilized mechanism of pathogen defense that also results in collateral damage to host tissues [15]. In addition, multiple pathogens have been shown to use ROS production as an important virulence determinant, suggesting that oxidative damage could be classified as a pattern of pathogenesis [28–31]. Regardless of the source of oxidative damage—bacterial attack, host defense, or xenobiotic assault—a critical response is the activation of detoxification enzymes that mitigate the damage. In addition, epithelial cells sensing oxidative stress would increase the survival of the host by initiating or amplifying local anti-pathogen defenses such as antimicrobial infection response gene expression, and endocrine signals that trigger an aversive learning program. Because FSHR-1 is required both for the survival of xenobiotic oxidative stress, and for the activation of oxidative stress response and infection response genes upon infection, we propose a model in which the FSHR-1 pathway could sense oxidative damage as a pattern of pathogenesis and then activate multiple facets of the innate immune response.

The ligand for FSHR-1 is unknown. FSHR-1 belongs to the LGR class of GPCRs; homologs bind the heterodimeric glycopeptide hormone FSHα/β [32–34]. The worm genome does not contain an identifiable FSHα subunit and no known role in immunity has been associated with the gene that most closely resembles FSHβ, suggesting that FSHR-1 may bind a noncanonical ligand. Because FSHR-1 is important for the defense against multiple dissimilar pathogens, it is not likely to be a PRR that binds a MAMP. We have shown that the FSHR-1 pathway functions in the absence of infection to promote survival of heavy metal and oxidative stress, suggesting that FSHR-1’s ligand must be endogenously produced rather than bacterial in origin. A recently described example of a GPCR that recognizes infection in *C*. *elegans* by binding an endogenous ligand is the DAMP receptor DCAR-1 [4]. HPLA is a tyrosine metabolite that is produced by the worm upon physical disruption of the cuticle and underlying hypodermis by a sterile wound or by the piercing hyphae of the pathogenic fungus *Drechmeria coniospora*. Rather than detecting physical damage as a sign of infection, it is tempting to speculate that FSHR-1 could bind an endogenously-produced DAMP that signals chemical damage caused by ROS production. An important future step in understanding the recognition and response to infection in epithelial cells is the identification of the FSHR-1 ligand.

## Methods

### *C*. *elegans* strains and maintenance

*C*. *elegans* were grown at 20°C on NGM agar plates seeded with *E*. *coli* strain OP50 as previously described [35]. For experiments requiring developmentally synchronized worms, gravid hermaphrodites were bleached to obtain embryos, which hatched in sterile M9 buffer overnight at room temperature. Arrested L1s were pipetted to the appropriate seeded plates to resume development.

### Killing assays

Infection-based “slow killing” pathogen assays were performed as described [36,37]. Briefly, 10 μl of a saturated overnight culture of *P*. *aeruginosa* PA14 was pipetted onto 3.5 cm SK plates (NGM, with 0.35% Bacto-peptone) and spread slightly (standard lawn) or to cover the plate (big lawn). The plates were incubated at 37°C for 24 hours, followed by 25°C for 24 hours [38]. FUDR (75 μg/ml) was added to the perimeter of the agar 30–60 minutes before assay set-up. L4 larvae were transferred to the plates, incubated at 25°C, and monitored for survival over time [36,37].

For oxidative and cadmium stress killing assays, 3.5 cm NGM plates were poured with a supplement of paraquat or CdCl_2_. Concentrations of paraquat between 0.2 mM and 200 mM are commonly used to induce cytoplasmic oxidative stress so we used 5 mM paraquat in our assays [39,40]. We used 100 μM cadmium to induce heavy metal stress, as previously described [41]. Plates were seeded with 20x concentrated OP50 and FUDR (75 μg/ml) was added to the perimeter of the agar 30–60 minutes before assay set-up. L4 larvae were transferred to the plates and monitored for survival over time using a nose-touch response assay.

For heat stress killing assays, synchronized L1s were pipetted onto NGM plates seeded with OP50 and grown at 20°C until the young adult stage. Plates were transferred to 37°C and the numbers of live and dead worms were scored at multiple time points using a nose-touch response assay. To minimize temperature fluctuations throughout the assay, each plate was scored only once and then discarded.

All killing assays were performed in triplicate with approximately 40 worms per plate, and each experiment was repeated at least three times. Mean survival time was calculated for each plate as described [37]. Conditions were compared using a one-way ANOVA and Tukey HSD test.

### Pathogen accumulation

To measure accumulation of pathogens, N2 wild-type or *fshr-1(ok778)* L4 worms were transferred to SK plates prepared as for a pathogen killing assay, but with GFP-labeled PA14. After 18 hours of incubation at 25°C, worms were anesthetized with 25 mM NaN_3_, then photographed using a Nikon 90i compound epiflourescence microscope. To quantify the pathogen accumulation, the sum of the fluorescence intensity in the worm was determined for at least 10 worms per condition.

### Avoidance assays

To measure bacterial avoidance, 7 μl of PA14 or 10 μl of OP50 saturated overnight culture was pipetted onto a 3.5 cm SK plate. The plates were grown at 37°C for 24 hours, followed by 25°C for 24 hours. L4 worms were transferred to the center of the bacterial spot and incubated at 25°C. All plates were scored for avoidance of the lawn at least 30 minutes but no more than 1 hour after set-up, and again 9 hours after set-up. Worms were scored as “in” the lawn if at least 50% of their posterior body were in contact with the lawn or if their head were in contact with and foraging in the lawn. Worms were scored as “out” of the lawn if their head and >50% of their body were outside the lawn. All experiments were performed in triplicate and repeated at least three times. The mean lawn occupancies for different conditions were compared using a one-way ANOVA and Tukey HSD test.

### Microarray

To measure genome-wide gene expression, young adult N2 wild-type or *fshr-1(ok778)* worms were transferred to SK plates with PA14 or OP50 prepared as if for a pathogen killing assay. Samples were prepared as previously described [12]. Briefly, total RNA was harvested in TriReagent (Molecular Research Center) after 4 hours of incubation on SK plates. cDNA was synthesized following the recommended RetroScript (Life Technologies) protocol. After second strand synthesis, double-stranded cDNA was purified using an RNeasy (Qiagen) column. Samples were hybridized on Affymetrix full-genome GeneChips for *C*. *elegans* at the Harvard Medical School Biopolymer Facility. Each experiment was repeated three times. The data were analyzed using GenePattern (Broad Institute) and Microsoft Excel. The mean expression for the three replicates of each gene was compared between conditions using a two-tailed two-sample t-test. A gene was considered differentially expressed between conditions if there was a greater than 2-fold difference in mean expression with P<0.05.

To validate the microarray data, quantitative RT-PCR was performed as previously described [12] on four genes that were induced in infected worms according to microarray data. Briefly, wild-type N2 and *fshr-1(ok778)* mutant young adults were transferred to SK plates with PA14 or OP50 prepared as if for a pathogen killing assay. After 4 hours, worms were washed off the plates with M9 buffer and washed two more times to remove excess bacteria. Total RNA was extracted using TRI-Reagent. cDNA was synthesized using a Retro-script kit (Ambion) with oligo-dT primers, and then amplified on a MyiQ2 iCycler (Bio-Rad) with iQ supermix containing SYBR green (Bio-Rad). Primers are available upon request. Normalized expression values were compared among three independent biological replicates. For each experiment, the induction was calculated as the expression in worms exposed to PA14 divided by the expression in worms exposed in parallel to OP50. A two-sample T-test was used to compare the induction in wild-type worms with the induction in *fshr-1(ok778)* mutants.

### Reporter induction

To measure induction of the *ldIs3 [gcs-1*::*gfp]* oxidative stress response reporter, L4 LD1171 *ldIs3* or JRP1024 *fshr-1(ok778); ldIs3* worms were transferred to SK plates with PA14 or OP50 prepared as if for a pathogen killing assay. After 5 hours, worms were anesthetized with 25 mM NaN_3_, then photographed using a Nikon 90i compound epiflourescence microscope. To quantify reporter expression, the intestine was manually outlined using the Nikon Elements Advanced Research software. The mean fluorescence intensity of this Region of Interest was determined by the software for at least 10 worms per condition.

To validate the microarray data, reporter strains BC11778 *sEx11778 [gpx-1*::*gfp]* and BC14266 *sEx14266 [gst-38*::*gfp]* [42] were transferred as L4 larvae to SK plates with PA14 or OP50 prepared as described above. After 5 hours, worms were anesthetized and photographed as described above. The exposure time was held constant for all conditions within an experiment.

## Supporting Information

S1 Fig Microarray validation using qRT-PCR. (A) Induction of a sample of genes in worms infected with PA14 for 4 hours relative to control worms grown in parallel on non-pathogenic OP50. Expression was measured using qRT-PCR, and induction was defined as the expression in worms on PA14 divided by expression in worms on OP50. (B) The induction of the same genes based on microarray data. Error bars are SEM. T-test comparison of induction in wild-type and *fshr-1(ok778)* mutants, *P<0.05, **P<0.01.
(TIF)

S2 Fig Microarray validation using transcriptional reporters. (A, B) Expression of the reporter *gpx-1*::*GFP* in worms grown on OP50 (A) or infected with PA14 for 5 hours (B). (C, D) Expression of the reporter *gst-38*::*GFP* in worms grown on OP50 (C) or infected with PA14 for 5 hours (D). (E) The expression of the same genes in worms grown on OP50 or infected with PA15, based on microarray data. Error bars are SEM. T-test comparison of microarray expression in wild-type worms fed OP50 or PA14, **P<0.01.
(TIF)

S3 Fig FSHR-1 is required for the avoidance of pathogenic PA14. L4 *fshr-1* (RNAi) and L4440 control (RNAi) worms were transferred to lawns of pathogenic *P*. *aeruginosa* PA14. The lawn occupancy was measured 1 hour and 9 hours after the transfer and the means of the two RNAi conditions were compared at each time point with a T-test. ***P<0.001. Error bars are SEM.
(TIF)

S1 Table Ratio of basal gene expression in uninfected worms. The ratio of gene expression was calculated as the expression in wild-type N2 worms / *fshr-1(ok778)* mutant worms grown on *E*. *coli* OP50. The genes shown have at least 2-fold higher expression in wild-type worms than in mutants, with P<0.05 in a two-sample T-test.
(XLSX)

S2 Table Ratio of gene expression in infected worms. The ratio of gene expression was calculated as the expression in wild-type N2 worms / *fshr-1(ok778)* mutant worms grown on *E*. *coli* OP50 until the young adult stage and then transferred to *P*. *aeruginosa* PA14 for 4 hours. The genes shown have at least 2-fold higher expression in wild-type worms than in mutants, with P<0.05 in a two-sample T-test.
(XLSX)
