# Supplementary figures and images for: The Conserved G-Protein Coupled Receptor FSHR-1 Regulates Protective Host Responses to Infection and Oxidative Stress
Source: PLoS One. 2015 Sep 11;10(9):e0137403. doi: 10.1371/journal.pone.0137403 (PMC4567296; doi:10.1371/journal.pone.0137403)

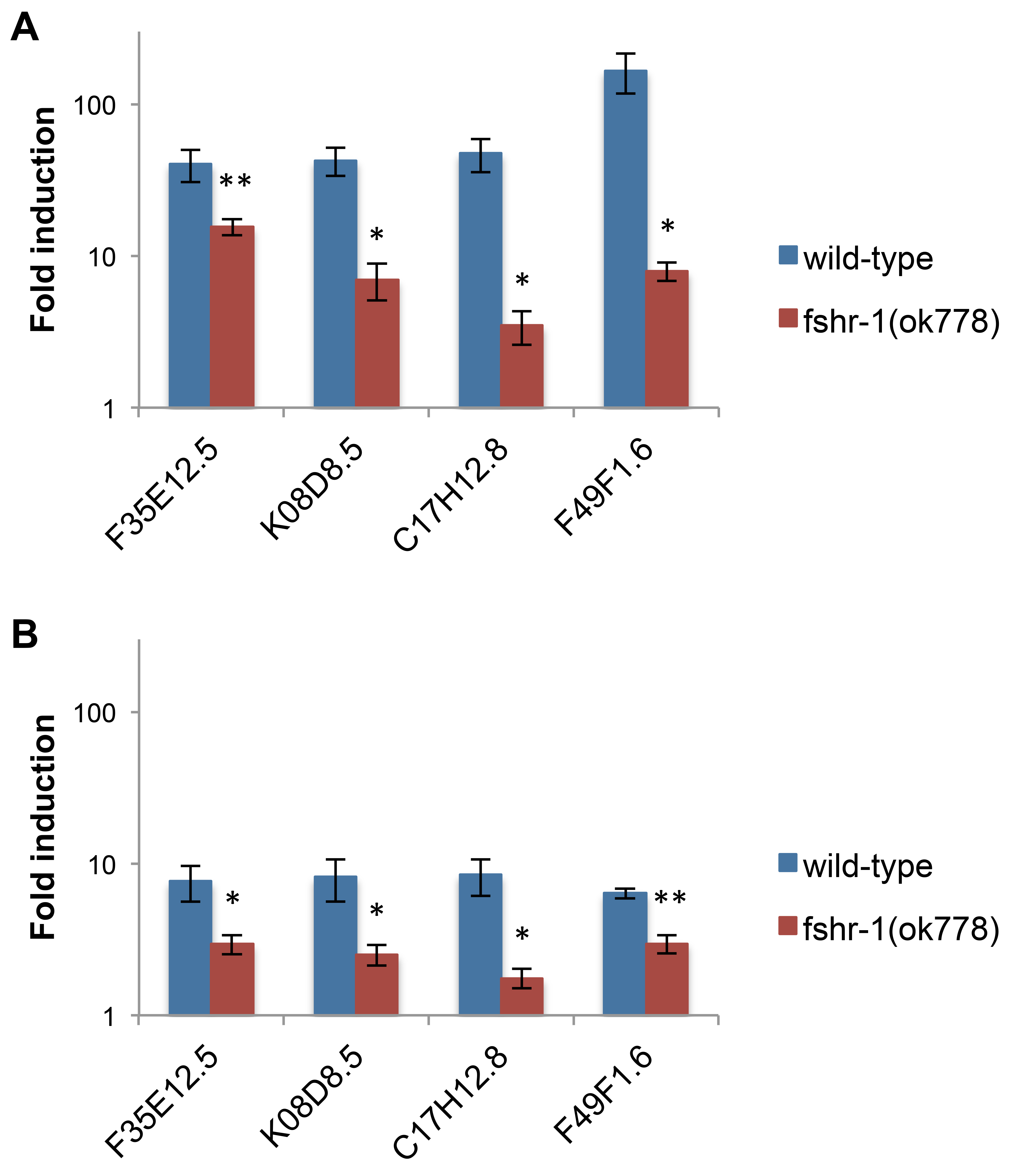

Supplement: S1 Fig — (A) Induction of a sample of genes in worms infected with PA14 for 4 hours relative to control worms grown in parallel on non-pathogenic OP50. Expression was measured using qRT-PCR, and induction was defined as the expression in worms on PA14 divided by expression in worms on OP50. (B) The induction of the same genes based on microarray data. Error bars are SEM. T-test comparison of induction in wild-type and fshr-1(ok778) mutants, *P<0.05, **P<0.01. (TIF) [file pone.0137403.s001.tif]

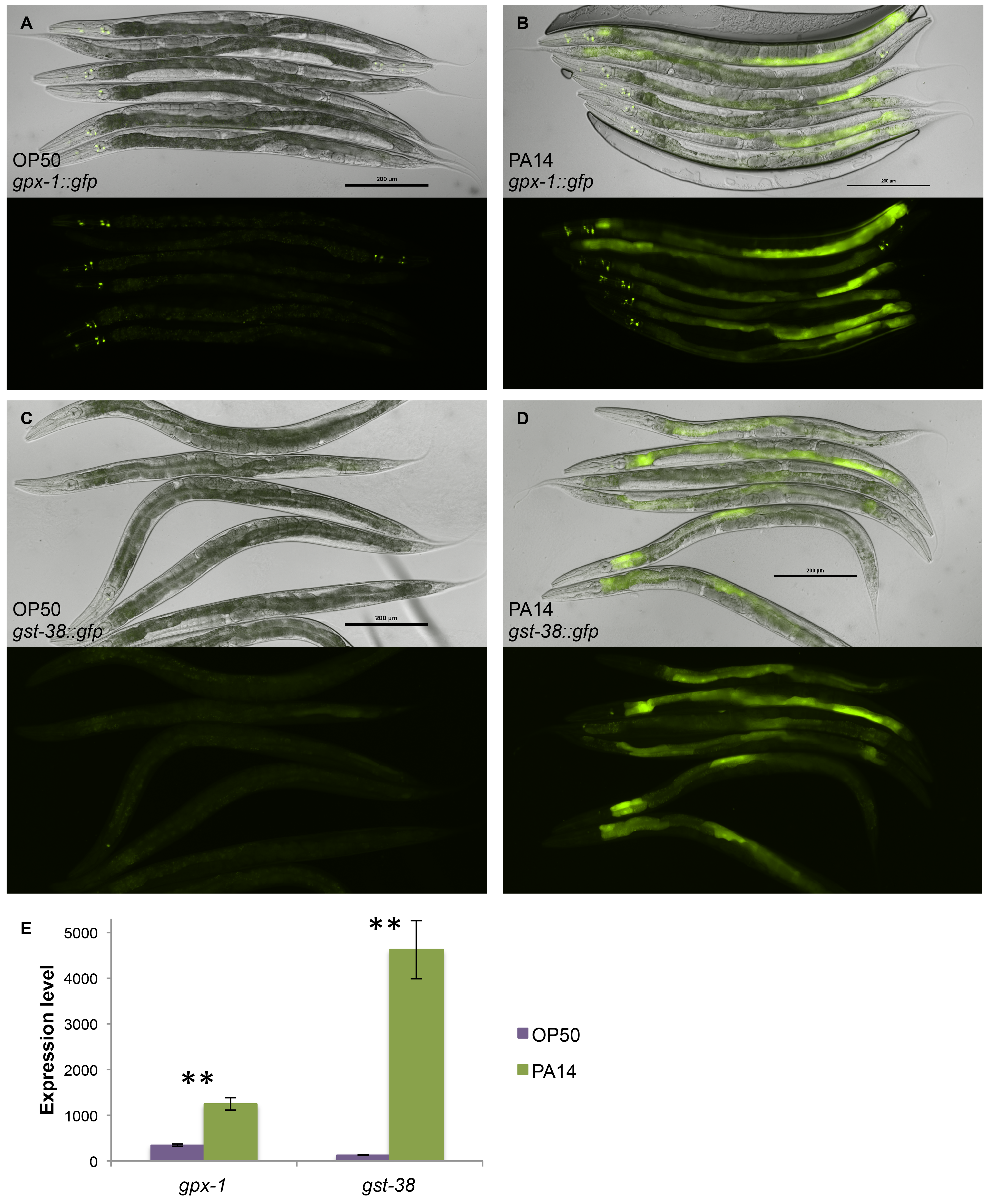

Supplement: S2 Fig — (A, B) Expression of the reporter gpx-1::GFP in worms grown on OP50 (A) or infected with PA14 for 5 hours (B). (C, D) Expression of the reporter gst-38::GFP in worms grown on OP50 (C) or infected with PA14 for 5 hours (D). (E) The expression of the same genes in worms grown on OP50 or infected with PA15, based on microarray data. Error bars are SEM. T-test comparison of microarray expression in wild-type worms fed OP50 or PA14, **P<0.01. (TIF) [file pone.0137403.s002.tif]

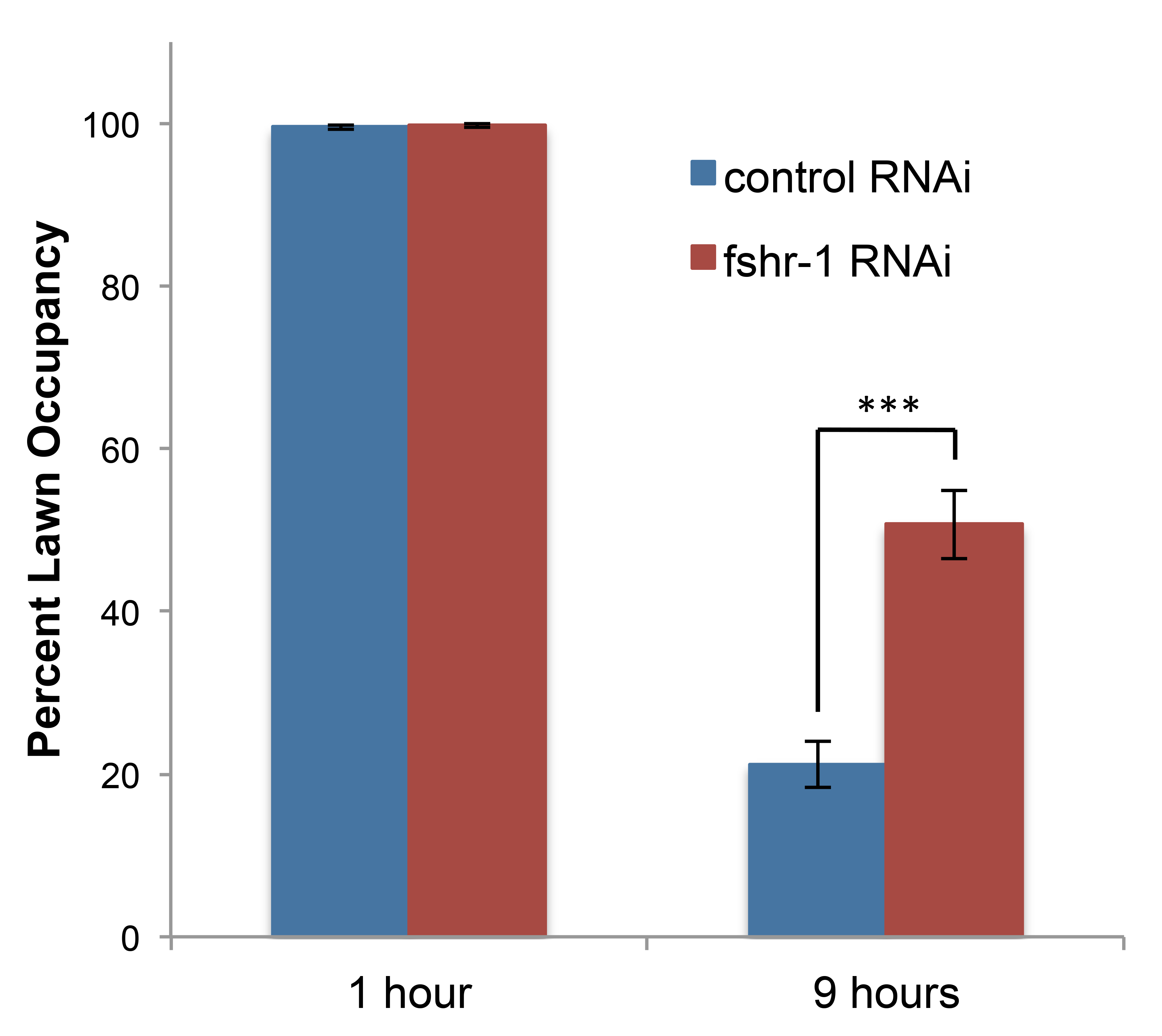

Supplement: S3 Fig — L4 fshr-1 (RNAi) and L4440 control (RNAi) worms were transferred to lawns of pathogenic P. aeruginosa PA14. The lawn occupancy was measured 1 hour and 9 hours after the transfer and the means of the two RNAi conditions were compared at each time point with a T-test. ***P<0.001. Error bars are SEM. (TIF) [file pone.0137403.s003.tif]
